# Reestablishment of p53/Arf and interferon-*β* pathways mediated by a novel adenoviral vector potentiates antiviral response and immunogenic cell death

**DOI:** 10.1038/cddiscovery.2017.17

**Published:** 2017-03-20

**Authors:** Aline Hunger, Ruan FV Medrano, Daniela B Zanatta, Paulo R Del Valle, Christian A Merkel, Thiago de Almeida Salles, Daniel G Ferrari, Tatiane K Furuya, Silvina O Bustos, Renata de Freitas Saito, Eugenia Costanzi-Strauss, Bryan E Strauss

**Affiliations:** 1 Viral Vector Laboratory, Center for Translational Investigation in Oncology, Cancer Institute of São Paulo/LIM 24, University of São Paulo School of Medicine, São Paulo, Brazil; 2 Centro de Bioterismo (Animal Care Center), University of São Paulo School of Medicine, São Paulo, Brazil; 3 Heart Institute (InCor), University of São Paulo Medical School, São Paulo, Brazil; 4 Natural Computing Laboratory (LCoN), Mackenzie University, São Paulo, Brazil; 5 Experimental Oncology Laboratory, Center for Translational Investigation in Oncology, Cancer Institute of São Paulo/LIM 24, University of São Paulo School of Medicine, São Paulo, Brazil; 6 Gene Therapy Laboratory, Biomedical Sciences Institute, University of São Paulo, São Paulo, Brazil

## Introduction

Malignant melanoma is a type of cancer with high death rates due, in part, to the lack of efficient treatments once metastases have formed.^1^ The tumor suppressor protein p53 is found in its wild-type form in 90% of melanoma cases, though other components of this pathway may be altered. Tolerance to p53wt during melanomagenesis can be achieved through the overexpression of its negative regulator HDM2, found in 56% cases,^2,3^ and loss of its functional partner on the CDKN2A locus (where p14ARF resides), which occurs in some 50% of primary melanomas.^4^ As p53wt is maintained at such a high frequency in melanoma, we and others propose that it may be recruited to participate in the therapeutic approach.^5–7^

Previously, we showed that introduction of the p53 functional partner, p19Arf (p19Arf in mice, p14ARF in humans) into B16 melanoma cells restored the p53 pathway and promoted cell death *in vitro* and *in vivo.*^8^ However, the impact of apoptotic cell death may be limited and is not likely to actively promote an antitumor immune response. Therefore, we sought to develop a cancer gene therapy approach that would induce cell death by a mechanism that would have wide-spread anti-cancer effects that reach beyond the treated cell.

Interferon-*β* (IFN*β*) is a pleiotropic cytokine with cytostatic, anti-angiogenic, pro-apoptotic and immunomodulatory activities^9^ and it interacts with the p53 pathway. For example, IFN*β* and p53 cooperate in antiviral defense^10–12^ while other works have implied that p14ARF is critical for induction of apoptosis in response to type I interferon.^13^ Together, these data suggest a promising benefit of using p19Arf in combination with IFN*β* for the induction of cell death.

Even high levels of cell death may lack the signaling necessary to promote an adaptive immune response that could then continue tumor killing at the site gene therapy and, possibly, at distant foci. Such signaling is associated with immunogenic cell death (ICD), including the exposure of calreticulin (CRT) and the release of ATP and HMGB1 by the dying cell, serving to stimulate the maturation of dendritic cells and elicit an efficient antitumor response.^14^ Many cellular pathways contribute to ICD, including endoplasmic reticulum stress, necroptosis and components of autophagy.^15–17^ The participation of type I interferon is also a key component of ICD^18^ and is a requirement for immune rejection of tumors,^19^ but the use of gene transfer for this purpose has not been widely examined.

Indeed, in our first attempt to explore the p19Arf and IFN*β* pathways, it was revealed that the combined delivery of this genes, but not individual applications, leads to an increase in B16 cell death *in vitro* and *in vivo* in a gene therapy model.^20^ Also, melanoma cells were transduced *ex vivo* and these dying cells were applied as an immunogen, conferring protection in a prophylactic vaccine model as well as tumor reduction in a therapeutic vaccine assay,^21^ but the mechanism of cell death was not explored in detail and thus the reason why the p19Arf+IFN*β*-treated cells respond with a superior immune stimulus remained unclear. In these previous works, we used adenoviral vectors with the wild-type fiber to transduce B16 cells with forced expression of the coxsackievirus and adenovirus receptor (CAR).^20,21^ Forced expression of CAR may alter cellular phenotype^22^ and broad application of our gene transfer approach was limited by the need for the CAR-mediated adenoviral transduction of target cells. Here we present for the first time the effects of a novel adenoviral vector with an RGD-modified knob protein for increased viral tropism. In this system, the p19Arf and IFN*β* transgene expression is controlled by a p53-responsive promoter, which was developed in our laboratory and confers higher-gene expression and superior antitumor activity when compared to the CMV promoter.^23,24^

Taking advantage of this novel delivery system, we revealed (i) the bystander effect of IFN*β* produced by transduced cells on the non-transduced cells, (ii) the mechanisms of cell death unleashed by p19Arf and IFN*β* gene transfer, involving the release of ICD molecules by dying cells and (iii) that the activation of p53 pathway, in the presence of an endogenous antiviral response elicited by IFN*β*, culminates in massive cell death. Together, these results provide new insights as to why combined, but not individual, transfer of p19Arf and IFN*β* leads to cell death associated with superior antitumor immune protection.

## Results

### Combined p19Arf and IFN*β* gene transfer enhances cell death *in vitro* and arrests tumor growth *in vivo* involving activation of the p53 pathway

The AdRGD-PG vectors were established by the introduction of the p53-responsive promoter, called PG, resulting in a vector platform where transgene expression would be controlled by p53. The set of AdRGD-PG vectors constructed includes three monocistronic vectors, containing the eGFP, p19Arf or murine IFN*β* cDNAs and a bicistronic vector containing the IRES element between the p19Arf and IFN*β* genes (Supplementary Figure S1). These RGD-containing vectors can successfully transduce CAR-negative B16 cells (Supplementary Figure S2A) and express the reporter gene eGFP in a p53-responsive manner (Supplementary Figures S2B and C). The expression of p19Arf and IFN*β* by the mono and bicistronic vectors was also confirmed (Supplementary Figures S3 and S4).

**Figure 1:**
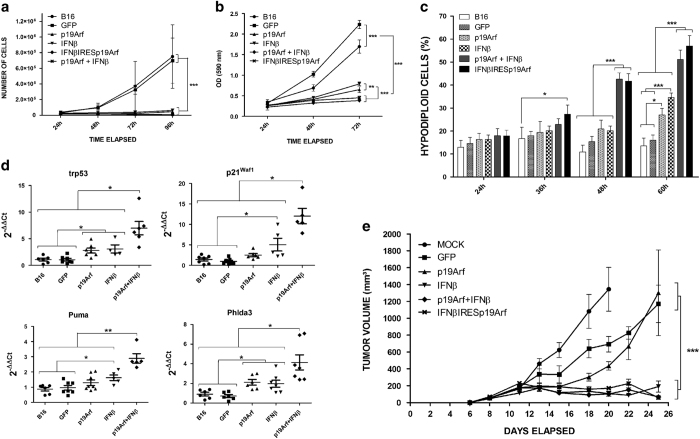
Gene transfer of p19Arf and IFN*β* induces accumulation of hypodiploid cells and p53 pathway genes. B16 cells were transduced with the AdRGD-PG vector and (**a**) replated for assessment of growth potential measured by counting viable cells or (**b**) replated in 96-well plates and quadruplicate samples were stained for MTT assay. (**c**) Cells were also analyzed by flow cytometry for evaluation of hypodiploid cells. Results from each graph in **a**, **b** and **c** represent the average and s.d. among at least duplicate samples from three independent experiments. **P<0.01 and ***P<0.001, two-way analysis of variance, followed by Bonferroni. (**d**) Mice were inoculated with B16 cells and treated with the AdRGD vectors. Tumors were collected for RT-qPCR analysis of trp53 pathway genes (*Trp53*, *p21^Waf1^*, *Puma* and *Phlda3*). *β*-actin and/or glyceraldehyde 3-phosphate dehydrogenase were used as the reference genes. Data represent the average and s.d. from duplicated PCR reactions derived from 7–10 animals per group. *P<0.05 and **P<0.01, one-way analysis of variance, followed by Kruskal–Wallis. (**e**) In parallel, mice were maintained for tumor size evaluation. Results represent the average and s.d. of tumor volumes (mm^3^). *N*=6 for all groups except Mock and GFP, where *N*=4. ***P<0.001, two-way analysis of variance, followed by Bonferroni.

Next, we observed the effects of gene transfer on B16 cells. Either p19Arf or IFN*β* reduced proliferation (Figure 1a), yet mitochondrial activity was reduced to a greater degree when the genes were combined (Figure 1b). We also observed that only combined treatment with p19Arf+IFN*β* resulted in altered cell cycle distribution (Supplementary Figure S5) and dramatically increased hypodiploid cells when compared to individual gene transfer (Figure 1c). Moreover, qPCR analysis of *Trp53* and its target genes (*p21^Waf1^*, *Puma* and *Phdla3*) revealed activation of this pathway especially upon combined treatment *in vitro* (Supplementary Figure S6).

The *in vivo* effect of combined p19Arf and IFN*β* gene transfer was verified in B16 tumors after *in situ* injections of AdRGD-PG vectors. Animals were accompanied for tumor growth or tumors were collected for evaluation of gene expression. As expected, p19Arf+IFN*β* treatment *in vivo* significantly increased transcript levels for *Trp53*, *p21^Waf1^*, *Puma* and *Phlda3* (Figure 1d) and significantly reduced tumor progression (Figure 1e). Note that even though IFN*β* treatment reduced tumor growth, lower or no increase in gene expression was observed in this condition as compared to p19Arf or controls. As shown here, the new AdRGD-PG vectors present a technological advantage, the treatment of CAR-negative cells.

### IFN*β* bystander effect and its enhancement in cells harboring p19Arf

**Figure 2:**
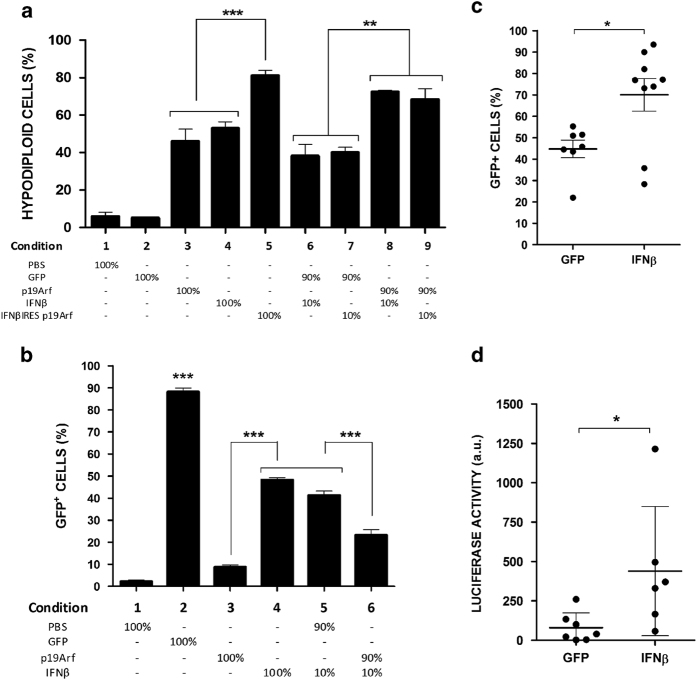
p19Arf can sensitize cells to IFN*β* bystander effect *in vitro* and *in vivo*. Cells were transduced with the indicated AdRGD-PG vectors and later mixed in the indicated proportions in six-well plates. (**a**) B16 cells were collected 72 h post transduction and accumulation of hypodiploid cells was revealed by flow cytometry. (**b**) B16ISRE-GFP-Luc cells were collected 48 h post transduction and the percentage of eGFP-positive cells was measured by flow cytometry. Results from each graph represent the average and s.d. from three independent experiments. *P<0.05, **P<0.01, ***P<0.001, one-way analysis of variance, followed by Tukey’s multiple comparison. For detection of the IFN*β* bystander effect *in vivo*, mice were inoculated with B16ISRE-GFP-Luc cells and treated with the AdRGD vectors as described in Figure 1. Tumors were collected 1 day after the last injection. (**c**) Detection of GFP indicates level of B16 transduction by AdRGD-PG-eGFP or bystander effect of AdRGD-PG-IFN*β* vectors. (**d**) Detection of luciferase activity reveals lack of bystander effect by AdRGD-PG-eGFP, but influence of AdRGD-PG-IFN*β*. Results represent the average and s.d. from tumors of at least six animals in each group. *P<0.05, *t*-test.

Although IFN*β* is a secreted protein, we hypothesize that even a small percentage of cells receiving AdRGD-PG-IFN*β* could have an impact on non-transduced neighboring cells. As seen in Figure 2a, when 10% of cells were transduced with AdRGD-PG-IFN*β*, the accumulation of hypodiploid cells was similar to that observed when 100% of cells were transduced with the IFN*β* vector. When we mixed cells transduced with the p19Arf adenoviral vector with cells transduced with the IFN*β* or the bicistronic vectors (9 : 1 proportion), the number of hypodiploid cells increased, suggesting that p19Arf can sensitize the cells and augment the bystander effect mediated by IFN*β*.

The cellular response to IFN*β* was confirmed using a stably modified B16 cell line where expression of both GFP and luciferase reporter genes is under control of the interferon-stimulated response element, ISRE (here after called B16ISRE-GFP-Luc). When transduced and co-cultivated as described above, we observed that 10% of cells transduced with AdRGD-PG-IFN*β* were sufficient to induce reporter gene expression in more than 40% of the population, essentially the same as seen when transducing 100% of the cells with AdRGD-PG-IFN*β* (Figure 2b). When p19Arf-transduced B16ISRE-GFP-Luc cells were co-cultivated with IFN*β*-producing cells, the induction of reporter gene activity was reduced, presumably due to cell death. No significant induction of ISRE was caused by transduction with AdRGD-PG-p19Arf. Treatment with the GFP adenoviral vector was used as a control to quantify the transduction efficiency. Together, these results indicate a strong bystander effect mediated by IFN*β* that is further enhanced in the presence of p19Arf.

The bystander effect of IFN*β* would be particularly interesting *in vivo* since we do not expect that all tumor cells are transduced upon gene therapy. To verify this, B16ISRE-GFP-Luc cells were inoculated in C57BL/6 mice and tumors were treated *in situ* with AdRGD-PG-eGFP or AdRGD-PG-IFN*β* vectors. By assaying GFP-positive cells 24 h later, we verified that AdRGD-PG-eGFP transduced only 45% of tumor cells, but the IFN*β* bystander effect is extended remarkably to 70% of cells (Figure 2c). Luciferase activity was also measured revealing induction of ISRE only when tumors were treated with the AdRGD-PG-IFN*β* vector (Figure 2d). These data demonstrate that the number of cells affected by IFN*β in vivo* can be much greater than the number of cells transduced by the adenoviral vector, indicating that the bystander effect broadens the impact of gene therapy at the site of treatment.

### p19Arf and IFN*β* gene transfer elicits an antiviral response in B16 cells

**Figure 3:**
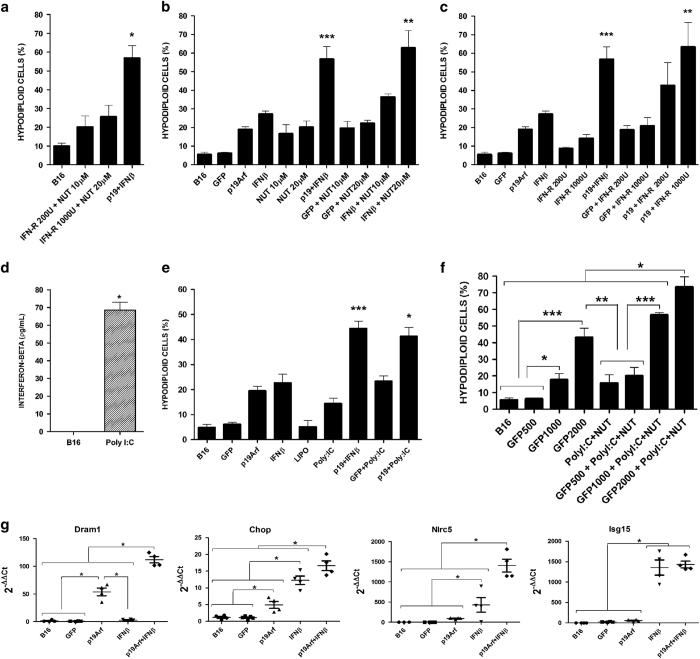
Cell death induced by p19Arf+IFN*β* treatment depends on transduction by adenoviral vectors. B16 cells were transduced with the AdRGD-PG vectors (MOI 500 or as stated in figure), treated with nutlin-3, IFN-R or transfected with Poly (I:C) and, 72 h after, collected for flow cytometric analysis of hypodiploid cells. (**a**) Combined pharmacologic treatment. (**b**) Use of nutlin-3 instead of the p19Arf vector. (**c**) Use of IFN-R instead of the IFN*β* vector. (**d**) Production of IFN*β* upon transfection with Poly (I:C) (0.2 *μ*g/ml) (*P<0.05, *t-*test). (**e**) Use of Poly (I:C) (0.2 *μ*g/ml) in place of IFN-R. (**f**) Contribution of vector to cellular response upon pharmacological treatment (Poly (I:C) 0.2 *μ*g/ml+nutlin-3 10 *μ*M). Results from each graph represent the average and s.d. from at least three independent experiments. *P<0.05, **P<0.01, ***P<0.001, one-way analysis of variance, followed by Tukey’s multiple comparison. (**g**) RNA was extracted from transduced cells (36 h) and RT-qPCR analysis of *Dram*, *Chop*, *Nlrc5* and *Isg15* was performed. *β*-Actin and/or glyceraldehyde 3-phosphate dehydrogenase were used as the reference genes. Data represent the average and s.d. from duplicate PCR reactions derived from five independent biological experiments. *P<0.05 one-way analysis of variance, followed by Kruskal–Wallis.

To better characterize the induction of cell death upon simultaneous stimulation of the p19Arf and IFN*β* pathways, we compared pharmacological approaches with gene transfer of p19Arf or IFN*β*. We used recombinant IFN*β* (IFN-R) protein instead of the AdRGD-PG-IFN*β* vector and nutlin-3, a compound which inhibits the interaction between MDM2 and p53,^25^ instead of the AdRGD-PG-p19Arf vector. As shown in Figure 3a, the accumulation of hypodiploid cells upon treatment with IFN-R+nutlin-3 (20–30%) was far inferior to that seen with p19Arf and IFN*β* gene transfer (60–65%). This result implies that our gene transfer approach is superior to pharmacologic treatment.

The combination of nutlin-3 with AdRGD-PG-IFN*β* provided significant levels of cell death (Figure 3b), suggesting that the critical role of p19Arf gene transfer is to inhibit p53–MDM2 interaction and that other functions of p19Arf are not essential here. Similarly, as shown in Figure 3c, treatment with IFN-R resulted in significant accumulation of hypodiploid cells only when combined with AdRGD-PG-p19Arf.

To confirm this result, we used the double-stranded RNA analog Poly (I:C), which is a toll-like receptor-3 agonist shown to induce IFN*β* production by B16 cells.^26^ As expected, Poly (I:C) treatment promoted IFN*β* production (Figure 3d) and, when associated with AdRGD-PG-p19Arf, induced similar levels of hypodiploid cells compared to combined gene transfer (Figure 3e).

Strikingly, combined Poly (I:C)+nutlin-3 treatment did not affect tumor cells, but did only in the presence of the AdRGD-PG-eGFP vector (Figure 3f). Cell death was induced in a multiplicity of infection (MOI) responsive manner, reaching a level comparable to that seen with AdRGD-PG-IFN*β*+AdRGD-PG-p19Arf treatment. Together, these data suggest that the gene transfer approach results in higher levels of cell death as compared to the use of IFN-R or Poly (I:C) together with nutlin-3 and that the success of the pharmacologic approach requires transduction with an adenoviral vector during simultaneous stimulation of both p53/MDM2/Arf and IFN*β* pathways.

Since the presence of the adenoviral vector is required, we evaluated the expression of genes related to antiviral response in B16 cells treated with p19Arf and IFN*β* gene transfer. We show that the transcripts of *Dram1*, a p53-responsive regulator of autophagy^27^ that can be induced by viral infection,^28,29^ *Chop*, an unfolded protein response-activated transcription factor involved in antiviral response^30^ and *Nlrc5*, a classical antiviral response gene,^31,32^ were all induced to higher levels in the presence of p19Arf+IFN*β* (Figure 3g). In addition, *Isg15* mRNA, another mediator of the antiviral response^33^ and a p53 target gene,^34^ was induced by IFN*β* gene transfer alone or in combination with p19Arf (Figure 3g). Together, these data imply that an endogenous antiviral response provoked upon adenovirus transduction in the presence of p19Arf and IFN*β* activity is a key component of our gene therapy approach.

In order to corroborate the antiviral response *in vivo*, tumors treated with *in situ* gene therapy were analyzed and an increase in *Isg15*, *Nlrc5*, *Dram1* and *Chop* gene expression was detected in cells treated with p19Arf or IFN*β* (Supplementary Figure S7A). Here the occurrence of autophagy *in vivo* was also verified by LC3*β* perinuclear staining only in cells treated with p19Arf+IFN*β* (Supplementary Figures S7B and C). These data show that the cell death mechanisms observed *in vitro* were preserved *in vivo*, including activities seen only upon combined p19Arf+IFN*β* gene transfer.

### p19Arf and IFN*β* gene transfer is associated with ICD

**Figure 4:**
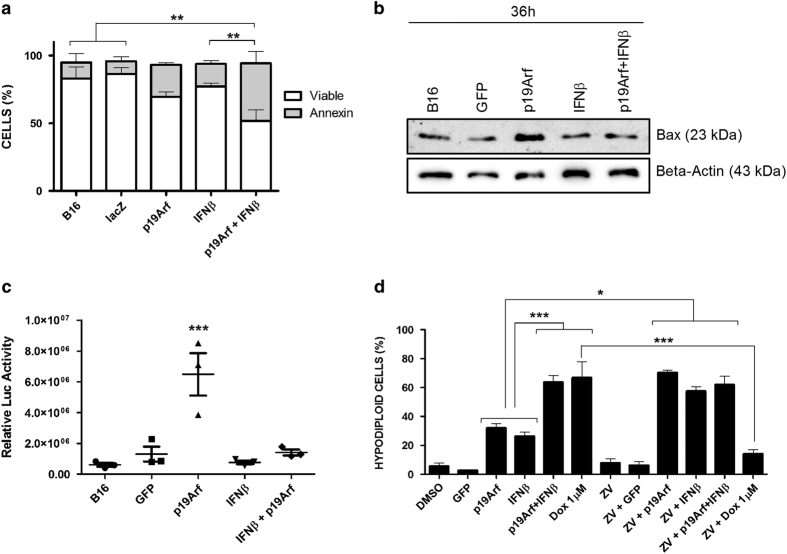
p19Arf gene transfer induces apoptosis but p19Arf+IFN*β* induces caspase-independent cell death. (**a**) Analysis of phosphatidylserine exposure by AnnexinV-PI double staining 72 h post transduction. Data represent the average and s.d. from three independent biological experiments **P<0.01 two-way analysis of variance, followed by Bonferroni (**b**) Bax expression detected by western blot analysis of protein extracts from B16 cells 36 h post transduction. (**c**) Caspase-3 activity as measured by luciferase reporter gene activity 36 h after transduction. (**d**) Treatment of cells with Z-VAD-FMK prior to treatment with adenoviral vectors indicates that p19Arf+IFN*β* induces cell death independent of caspase activity. Data (**c**, **d**) represent the average and s.d. from at least three independent biological experiments *P<0.05, ***P<0.001, one-way analysis of variance, followed by Tukey’s multiple comparison.

Next, we further explored the cell death mechanism elicited by our gene therapy approach. Even though treatment with p19Arf+IFN*β* conferred an increase in Annexin V-positive cells (Figure 4a), the expression of Bax (Figure 4b) and the activity of caspase-3 (Figure 4c), seen by a luciferase reporter vector,^35^ were surprisingly lower when compared to p19Arf treatment. In this regard, treatment of B16 cells with the pan-caspase inhibitor Z-VAD-FMK did not reduce cell death triggered by p19Arf+IFN*β* (Figure 4d). Surprisingly, Z-VAD-FMK treatment prior to transduction with p19Arf or IFN*β* increased the hypodiploid population (Figure 4d), pointing to a caspase-independent mechanism of cell death.

**Figure 5:**
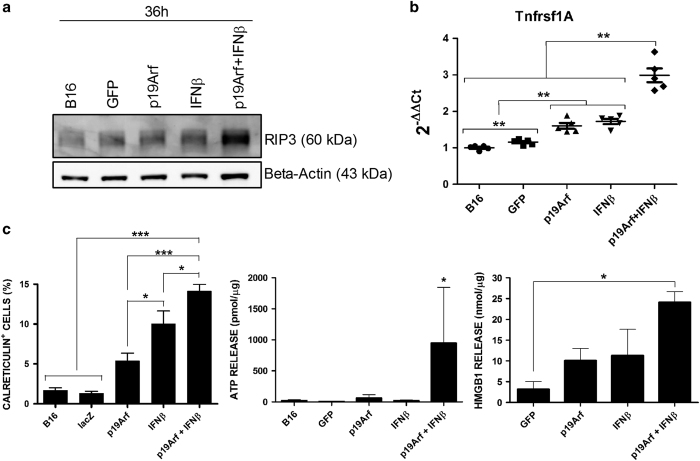
Gene transfer of p19Arf+IFN*β* induces features of necroptosis and ICD. (**a**) Protein levels of RIP3 were evaluated from B16 extracts and were increased upon p19Arf+IFN*β* treatment. (**b**) Total RNA extraction and RT-qPCR analysis of cells treated with AdRGD-PG vectors show an increase in TNFRSF1A (TNFR) expression after p19Arf+IFN*β* treatment. *β*-Actin and/or glyceraldehyde 3-phosphate dehydrogenase were used as the reference genes. Data represent the average and s.d. from duplicated PCR reactions derived from five independent biological experiments. **P<0.01 one-way analysis of variance, followed by Kruskal–Wallis. (**c**) Cells were collected for flow cytometric analysis of CRT exposure and supernatant was collected for measure of secreted ATP or HMGB1 release by B16 cells. Graphs represent the average and s.d. from at least three independent experiments. *P<0.05, ***P<0.001, one-way analysis of variance, followed by Tukey’s multiple comparison.

Necroptosis was originally identified as an alternative cell death program activated when caspase was blocked and is now also recognized as cellular defense mechanism against infection, including dsDNA viruses.^36,37^ Expression of both RIP3 (Figure 5a), the key mediator of necroptosis^38^ and the TNF receptor (*Tnfrsf1a*, Figure 5b), an activator of the necrosome complex,^38^ were increased only in cells treated with p19Arf+IFN*β*.

Recently, necroptotic cells have been shown to undergo ICD upon chemotherapy.^39^ Similarly, as observed in Figure 5c, only combined gene transfer resulted in the increase of all three markers of ICD, CRT exposure, secretion of ATP and release of HMGB1, as compared to the other conditions. Taken together, these data indicate that p19Arf+IFN*β* treatment induces a cell death mechanism with features of necroptosis and culminates in ICD.

### Molecular pathways and cellular functions associated with p19Arf and IFN*β* treatment in B16 cells

**Figure 6:**
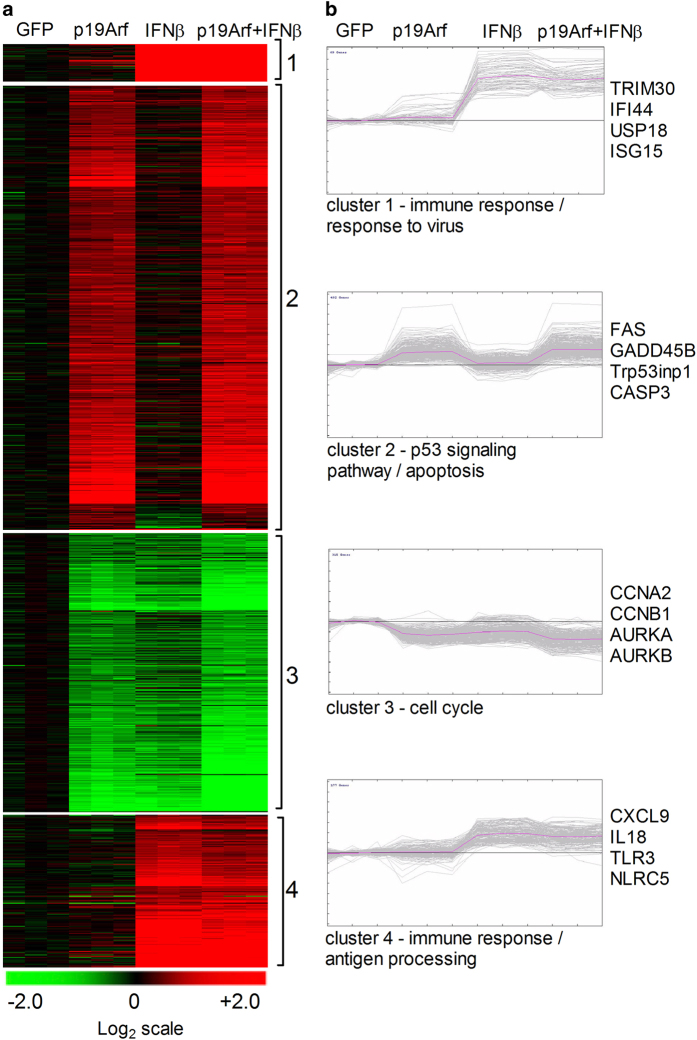
Genome-wide transcriptome analysis of p19Arf and IFN*β*-treated cells. (**a**) K-means clustering shows heatmap of transcript levels for the four clusters (log2-tranformed expression intensities); (**b**) Selected K-means clusters showing genes involved in different functions. Cluster 1 represents genes that are upregulated in IFN*β* and p19Arf+IFN*β* treatments, cluster 2 represents genes that upregulated in p19Arf and p19Arf+IFN*β* treatments, cluster 3 are genes downregulated in all treatments compared to GFP and cluster 4 represents genes that are upregulated in IFN*β* and p19Arf+IFN*β* treatments, but with less intensity.

To further elucidate the pathways that underlie cell death induced by p19Arf and IFN*β* treatment, whole-genome transcriptome analysis was performed. The unsupervised hierarchical clustering of samples was distinct for each of the conditions (GFP, p19Arf, IFN*β* and p19Arf+IFN*β*; Supplementary Figure S8) and K-means analysis revealed that four gene clusters and pathways are modulated according to the different treatments (Figure 6a). Genes involved in immune response, response to virus and antigen processing were strongly upregulated with IFN*β* and p19Arf+IFN*β* treatments (clusters 1 and 4 – additional file chart_cluster 1 and 4), evidenced by the genes *Trim30*, *Ifi44*, *Usp18*, *Isg15*, *Cxcl9*, *Il18*, *Tlr3* and *Nlrc5*, for example. On the other hand, genes involved in the p53 signaling pathway and apoptosis are upregulated by p19Arf and p19Arf+IFN*β* treatments (cluster 2 – additional file chart_cluster 2), enriched by *Fas*, *Gadd45b*, *Trp53inp1* and *Casp3* genes. Molecular pathways involved in cell cycle control indicate that this function is being strongly affected by p19Arf, IFN*β* or p19Arf+IFN*β* treatments (cluster 3 – additional file chart_cluster 3), as indicated by the downregulation of *Ccna2*, *Ccnb1*, *Aurka* and *Aurkb* genes, among others (Figure 6b).

These results provide a molecular framework for the induction of cell death triggered by the reestablishment of the apoptotic p53/Arf pathway in the presence of an IFN*β* antiviral response, demonstrating that cell death is a result of cooperative functions between these two pathways.

**Figure 7:**
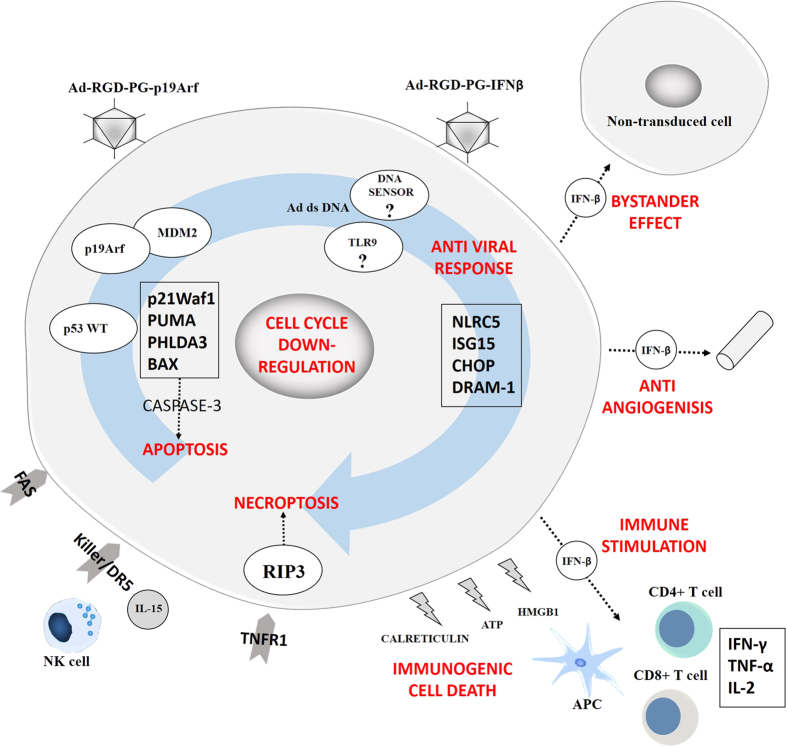
Schematic representation of the proposed mechanism of action of combined p19Arf and IFN*β* gene therapy in B16 cells.

Based on the data presented here and in our previous studies, we propose the following mechanism (Figure 7). First, reintroduction of p19Arf blocks MDM2 function and frees p53 to activate its pro-apoptotic pathway, as demonstrated by upregulation of p53 pathway genes, increase in caspase-3 activity and Bax expression.^20^ However, the cell death stimulus (blue arrow) of the p19Arf treatment is not strong enough to provoke high levels of cell death. At the same time, upon treatment with IFN*β*, an antiviral defense mechanism is activated, evidenced by the microarray analysis, which allows B16 cells to detect adenovirus components, most likely dsDNA in the cytoplasm through the DAI dsDNA sensor. In this regard, without the presence of the adenovirus, the activation of p19Arf and IFN*β* pathways is not enough to induce death. However, when the p53/Arf and antiviral IFN*β* pathways are combined, a cooperative context is achieved and the stimulus is strong enough to provoke massive cell death. This cellular death process displays features of necroptosis, suggested by RIP3 expression, upregulation of the TNF receptor and the absence of caspase-3 activity. On the other hand, in the extracellular environment, ICD markers (ATP, CRT and HMGB1) are released along with the secretion of IFN*β* to promote an antitumor immune response, resulting in the activation of NK cells, CD4+ and CD8+ T lymphocytes and immune protection to the host, as seen in our previous work.^21^ As shown here, IFN*β* can also affect non-transduced cells trough a bystander effect and possibly, due to its established anti-angiogenic activity, can affect the surrounding blood vessels as well.

## Discussion

The work presented here elucidated several molecular and cellular mechanisms induced by the adenovirus-mediated gene transfer of p19Arf together with IFN*β* that culminates in ICD of B16 melanoma cells.

We describe the utilization of a vector platform modified to include the RGD tripeptide in its fiber, allowing for the efficient transduction in a wide range of target cells without dependence on the Ad5 receptor, CAR. In addition, a bicistronic vector was constructed which contains the combination of therapeutic genes, ensuring the transfer of both factors to the target cells at the same time.

The influence of IFN*β* bystander effect on non-transduced cells was confirmed *in vivo*, yet the presence of p19Arf-sensitized B16 cells to IFN*β in vitro*, showing that the antitumor effects of our treatment extend beyond the transduced cells, possibly affecting a variety of cell types in the tumor microenvironment. To the best of our knowledge, this is the first report showing the extent of the IFN*β* bystander effect upon *in situ* gene therapy mediated by an adenoviral vector.

*In situ* application of our p19Arf+IFN*β* therapy effectively inhibited tumor growth correlating with the upregulation of *Trp53*, *p21^Waf1^*, *Puma* and *Phlda3* mRNA levels. Even though IFN*β* by itself could also inhibit tumor growth, our previous observations indicate that only p19Arf+IFN*β* treatment confers superior immune stimulation involving the NK cells, CD8+ and CD4+ T cells.^21^ Here we have evidenced the release all three classic markers of ICD (CRT, ATP and HMGB1), providing a likely mechanism for the results seen in our previous study.

Interactions of p53/Arf and IFN*β* pathways have been shown to play a role in controlling virus infection including through the induction of cell death.^11,12^ Here we have shown that the presence of the adenoviral vector and the activation of the p53/Arf and IFN*β* pathways are critical for the induction of high levels of cell death. Indeed, IFN*β* only induced significant cell death with the concomitant inhibition of MDM2 and in the presence of the viral vector. These observations were further supported by the induction of the *Isg15*, *Nlrc5*, *Dram* and *Chop* genes, involved in antiviral response.^33,40,41^ Moreover, transcriptome analysis of genes activated by IFN*β* also revealed a clear antiviral molecular signature, thus providing support to our proposed model.

Innate antiviral responses to adenovirus are initiated upon the interaction of the RGD motif with *α*v integrins. After being internalized, adenoviral dsDNA can be sensed within the endosome via TLR9 and in the cytoplasm by the DNA-dependent activator of IFN-regulatory factors (DAI) and by NOD-like receptors (NLRs).^42^ Interestingly, DAI induces an IFN response^43,44^ and is able to sensitize cells to virus-induced necroptosis by directly activating RIP3 without the involvement of RIPK1.^37^ Given our observation that RIP3 was induced by p19Arf+IFN*β* gene therapy, an ideal context for the induction of necroptosis seems to be met when using our adenoviral vector for the transfer of these genes. Indeed, a recent report in the literature showed that RIP3 is critical for the induction of ICD, since dying cancer cells deficient in RIP3 were not able to induce an immune response in mice.^39^

In conclusion, using the novel AdRGD-PG vectors, the combined p19Arf and IFN*β* gene therapy approach induces high levels of cell death, summons the immune system to the battle and, as a result, is predicted to have a wide-spread impact on tumor inhibition. The mechanism of cell death includes reactivation of the p53/Arf apoptotic pathway, induction of innate antiviral response and ICD with involvement of necroptosis. While much development is required, we propose that the combined gene transfer approach of IFN*β* with p14ARF will bring superior clinical benefit not seen with IFN*β* biochemotherapy or gene therapy strategies that are only focused in killing tumor cells without activating an immune response.

## Materials and Methods

### Cell culture and lines

The adenovirus-transformed, human embryonic kidney cell line 293A (HEK293A, Invitrogen, Carlsbad, CA, USA) was maintained in DMEM (Invitrogen) supplemented with 10% bovine calf serum (HyClone, Logan, UT, USA), 100 *μ*g/ml gentamicin, 50 *μ*g/ml ampicillin and 2.5 *μ*g/ml fungizone, at 37 °C, in a humidified atmosphere of 5% CO_2_. The mouse melanoma cell line B16F10 (B16, ATCC CRL-6475, confirmed presence of mouse short-tandem repeats and the MART1, S100A1, SOX10 and TYR markers of melanoma, data not shown) was cultured as above, except using Roswell Park Memorial Institute medium (Invitrogen). B16mCAR cells have been described previously.^20^ The lentiviral caspase-3 reporter vector, which encodes a constitutively expressed luciferase-GFP protein separated from a polyubiquitin domain via a caspase-3 cleavage site,^35^ was obtained from Chuan-Yuan Li (Department of Radiation Oncology, University of Colorado School of Medicine, Aurora, CO, USA). The pGreenFire1-ISRE reporter construct, a lentiviral vector which expresses both GFP and luciferase in response to type I IFN signaling, was obtained commercially (System Biosciences, Mountain View, CA, USA). These vectors were used to transduce B16 cells. B16 cells transduced with caspase-3 reporter vector were selected for puromycin resistance (0.5 *μ*g/ml) and B16 cells transduced with pGreenFire1-ISRE were sorted (two rounds), based on GFP expression, resulting in the B16ISRE-GFP-Luc cell line.

### Construction of adenoviral vectors

Our group has a set of adenoviral vectors constructed following Gateway technology (Invitrogen), as described previously.^20^ In this system, we constructed desired expression cassettes in an entry vector (denominated pENTR) and, by site-directed recombination, transfer these cassettes to adenoviral vectors (denominated pAd/PL-DEST). We received from Dr Hiroyuki Mizuguchi (Osaka University, Japan) a modified adenoviral vector containing the RGD motif (between residues threonine-546 and proline-547 of the fiber protein) that follows Adeno-X technology (Clontech, Mountain View, CA, USA).^45^ In order to render this vector compatible with the site-directed recombination strategy and our existing pENTR vectors, extensive modifications were made. First, we removed the SV40, polyA and CMV sequences from pShuttle2 (Clontech) and then we inserted, between *Not* I sites in this vector, the Gateway sequence that was amplified from pAd/PL-DEST. Then, the Gateway sequence was removed from the new pShuttle vector and inserted in AdRGD vector, by *PI-Sce* I and *I-Ceu* I digestions. Once this was made, we could transfer, by site-directed recombination between pENTR (Invitrogen) and the new AdRGD-DEST vector, our expression cassettes containing the PG promoter, transgene of interest and a polyA sequence. The set of AdRGD-PG vectors constructed includes three monocistronic vectors, containing the eGFP or the p19Arf or the murine IFN*β* genes, and one bicistronic vector, which contains both p19Arf and IFN*β* genes separated by the IRES sequence described by Ghattas *et al.*^46^ All vectors express the transgenes under control of a p53-responsive promoter, called PG, previously described by Bajgelman and Strauss.^23^ Cloning details are available upon request.

### Adenovirus production

Virus production was performed as described previously.^47,48^ The biological titer was determined using the Adeno-X Rapid titration kit (Clontech). For transduction of target cells, the MOI was calculated based on the biological (infectious) titer.

### *In vitro* assays

To maintain brevity, detailed procedures for several assays (X-Gal staining, immunofluorescence detection of p19Arf, enzyme-linked immunosorbent assay for detection of IFN*β*, flow cytometric assessment of cell cycle, AnnexinV/PI staining, flow cytometric detection of eGFP, growth curve and cell viability assay) will not be presented here since they have been described previously,^8,20,21,24^ though additional information is available upon request.

### *In situ* treatment of B16 tumors using the adenoviral vectors

C57BL/6 mice (7 week old, female) were obtained from the Centro de Bioterismo, FMUSP and were maintained in SPF conditions, with food and water *ad libidum*. One million B16 cells (parental, non-modified) were inoculated in the left flank of C57BL/6 mice and, after establishment of the tumor (10 days), mice were treated with three intratumoral injections of 5×10^8^ infectious units of AdRGD-PG vectors in a volume of 25 *μ*l, every 2 days. Tumor size was determined by measurement with a digital caliper or mice were killed and tumors were collected 48 h after the last injection. Tumors were fixed for 24 h in 4% PFA, immersed in 30% sucrose and frozen before tissue sectioning with a cryostat and immunofluorescence detection of LC3*β* or RNA extraction. For RNA extraction, 100 mg of samples were lysed with a digestion buffer (200 mM TRis-HCl, 200 mM NaCl, 1.5 mM MgCl_2_, 2% SDS and 500 *μ*g/ml of proteinase K, pH 7.5) at 60 °C overnight and RNA were extracted by Trizol reagent (Invitrogen) following the fabricant’s instructions. RT-qPCR reactions were performed as described later.

In other experiment, 1×10^6^ B16ISRE-GFP-Luc cells were inoculated in the left flank of C57BL/6 mice and mice were treated with AdRGD-PG-eGFP or AdRGD-PG-IFN*β* vectors, as described above. One day after the last injection, mice were killed and tumors were collected for dissociation with RPMI containing 35 *μ*g/ml of liberase (Roche, Mannheim, Germany) and fixed with 4% PFA for cytometric analysis of GFP expression or lysed for analysis of luciferase expression, measured with Dual-Glo Luciferase Assay System (Promega, Madison, WI, USA) following the manufacturer’s instructions and using microplate reader Victor (Perkin-Elmer, Waltham, MA, USA).

All procedures and conditions were approved in accordance to the guidelines of animal care and use by the Scientific and Ethics Committee of the Cancer Institute of São Paulo, University of São Paulo School of Medicine.

### Analysis of IFN*β* bystander effect *in vitro*

In the co-culture experiment, B16 or B16ISRE-GFP-Luc cells were transduced as described for the growth curve and replated in six-well dishes with 1×10^5^ cells total. For the mixtures, we combined in the same dish 1×10^4^ cells transduced with one virus plus 9×10^4^ cells transduced with a different virus, achieving a proportion of 1 : 9 of the mixture. For controls, we plated 1×10^5^ cells of each transductions made previously. Cells were collected 72 h after the transductions for analysis of cell cycle or GFP expression by flow cytometry (FACScan, Becton Dickenson, San Jose, CA, USA).

### Treatment with nutlin-3, IFN-R and Poly (I:C)

Cells were plated 1×10^5^ cells/well in six-well dishes, transduced the next day with a MOI of 500 of the virus and/or treated with nutlin-3 (Sigma-Aldrich, St Louis, MO, USA), recombinant mouse IFN*β* (R&D Systems, Minneapolis, MN, USA) and/or Poly (I:C; Sigma-Aldrich). For treatment with Poly (I:C), cells were transfected using lipofectamine 2000 (Life Technologies, Carlsbad, CA, USA) following the manufacturer’s instructions. Cells were collected 72 h after transduction for cytometric analysis of cell cycle alterations. In parallel, supernatant of cells transfected with Poly (I:C) was collected for detection of IFN*β* by ELISA, as described elsewhere.^20^

### Reverse transcriptase quantitative PCR

Cells were plated 1×10^5^ cells/well in six-well dishes, transduced the next day with a MOI of 500 of the virus and collected 36 h later with 0.5 ml of Trizol reagent (Invitrogen) using a cell scraper. Total RNA was extracted following the fabricant’s instructions and RNA concentration was determined by measuring absorbance at 260 nm. Extracted RNA quality was accessed by the protein and salt concentration, and by visualizing the 18 and 28S ribosomal RNA bands in a 1% agarose gel. Primers (Supplementary Table S1) were designed on the basis of the coding region, using the software Primer Blast (https://www.ncbi.nlm.nih.gov/tools/primer-blast/). Primers are complementary to sequences present in different exons (separated by introns), as predicted in sequences deposited at http://www.ncbi.nlm.nih.gov/nucleotide. Two different reference genes were tested (*β*-actin and glyceraldehyde 3-phosphate dehydrogenase), and *β*-actin was determined to be the most stable among the samples. Efficiency of all designed primers was validated, being at minimum close to 100%. Total RNA (2 *μ*g) was reverse transcribed using random primers and moloney murine leukemia virus reverse transcripatse (Invitrogen). Reaction conditions were 100 ng cDNA (final volume of 10 *μ*l); 12.5 *μ*M of each primer; 5 ml of Syber Green PCR Master Mix (Invitrogen). Amplification conditions consisted of denaturation at 95 °C for 15 min, followed by 40 cycles denaturation at 95 °C for 15 s, annealing at 60 °C for 1 min and extension at 72 °C for 1 min. All samples were tested in duplicate and analyzed by the 7500 Fast Software, version 2.05 (Applied Biosytems, Foster City, CA, USA). The 2^−ΔΔCt^ method was used for gene expression quantification, and data are presented as compared with the non-transduced B16 condition.

### Western blot

Cells were plated 1×10^6^ cells in 100 mm dishes, transduced the next day with a MOI of 500 of each virus and collected 36 or 60 h after transduction and subjected to western blotting, as described previously.^20^ The blotted membranes were blocked with 5% non-fat milk for 1 h at room temperature and probed with the following antibodies: anti-PARP #9542 diluted at 1 : 1000 (Cell Signaling, Danvers, MA, USA), anti-Bax #sc-23959 diluted at 1 : 500 (6A7, Santa Cruz Biotechnology Europe, Heidelberg, Germany) and anti-RIP3 #sc-47364 diluted at 1 : 500 (C-16, Santa Cruz Biotechnology Europe). For the secondary antibody, a horseradish peroxidase-conjugated antibody was used and revealed using the ECL detection kit (GE Healthcare Life Sciences, Marlborough, MA, USA).

### Flow cytometric analysis of CRT exposure

Cells were plated 1×10^5^ cells/well in six-well dishes, transduced the next day with a MOI of 500 of the virus and collected after 72 h for immunostaining with a CRT-specific antibody (#NB300-545, Novus Biologicals, Littleton, CO, USA). After cells were washed with PBS and stained with Alexa488-conjugated anti-rabbit secondary antibody (Thermo Fisher, Rockford, IL, USA) for analysis by flow cytometry (FACScan, Becton Dickenson).

### Measurement of released ATP by cells

Cells were plated 1×10^5^ cells//well in six-well dishes, transduced the next day with a MOI of 500 of the virus and supernatant was collected 72 h after for ATP measurement. All cells (adherent and in supernatant) were collected in order to determine total protein concentration by Bradford assay with BSA standard. ATP concentration was measured by ENLITEN ATP Assay System (Promega) and normalized with protein concentration of the sample. Bioluminescence detection was made with microplate reader Victor (Perkin-Elmer).

### Measurement of HMGB1 release

Cells were plated 1×10^5^ cells/well in six-well dishes, transduced the next day with a MOI of 500 of the virus and supernatant was collected 72 h after for HMGB1 measurement as described by HMGB1 ELISA kit (IBL International GMBH, Hamburg, Germany). All cells (adherent and in supernatant) were collected in order to determine total protein concentration by Bradford assay with BSA standard and HMGB1 concentration was normalized with protein concentration of the sample.

### Immunofluorescence detection of LC3*β*

Tumor sections were blocked with bovine serum albumin, probed with a polyclonal antibody for LC3*β* #sc-16755 diluted 1 : 500 (N-20, Santa Cruz Biotechnology Europe) followed by an anti-goat secondary antibody labeled with Alexa-594 diluted 1 : 3000 (Molecular Probes, Eugene, OR, USA). Nuclear staining was performed with Hoechst 33258, 20 *μ*g/ml (Molecular Probes). Cells were visualized by confocal microscopy at ×100 amplification.

### Microarray analysis

Cells, 8×10^5^, were plated in 100 mm dishes and transduced 24 h later with a MOI of 500 of AdRGD-PG vectors in 4 ml of RPMI 10% FBS medium. Four hours later, 6 ml of RPMI 10% FBS medium were added and cells were maintained for additional 32 h. Then, adherent cells and cells in suspension were collected for RNA extraction. RNA extraction was performed using TRIzol reagent (Thermo Scientific, Waltham, MA, USA, 15596026) following the manufacturer’s instructions. RNA integrity was assessed using a Bioanalyzer 2100 (Agilent Technologies, Santa Clara, CA, USA, G2939AA). All the samples that had a RIN equal to or greater than eight were profiled on microarrays. Gene Chip Mouse 1.0 ST (Affymetrix Inc., 901171) was used according to the manufacturer’s instructions to determine gene expression. Microarray results from three biological experiments were analyzed using TM4 Microarray software suite (Dana-Farber Cancer Institute, Boston, MA, USA). Differential gene expression profile was obtained by comparing the p19Arf, IFN*β* and p19Arf+IFN*β* to eGFP-treated cells using the significance analyses for microarray (false discovery rate <1%). K-means clusters were designed using Euclidian distance. Hierarchical cluster was designed using Euclidian distance and complete linkage. Bootstrap was used to evaluate dendrogram’s consistency. Enrichment analysis was performed in DAVID database (EASE score <0.05). Data can be accessed through Annotare repository (http://www.ebi.ac.uk/arrayexpress/experiments/E-MTAB-5399).

### Statistical analysis

The GraphPad Prism 5 software (La Jolla, CA, USA) was used for statistical analyses. All comparisons were conducted through one-way analysis of variance, followed by Kruskal–Wallis or Tukey’s multiple comparison; two-way analysis of variance, followed by Bonferroni; or *t*-test. *P*-value lower than 0.05 was considered significant.
