# Supplementary material for: Reestablishment of p53/Arf and interferon-β pathways mediated by a novel adenoviral vector potentiates antiviral response and immunogenic cell death
Source: Cell Death Discov. 2017 Mar 20;3:17017–. doi: 10.1038/cddiscovery.2017.17 (PMC5357668; doi:10.1038/cddiscovery.2017.17)
Supplement: Supplementary Information [file cddiscovery201717-s5.pdf]

Table S1: Primers used for RT-qPCR.

Gene	Primer (5'>3')	
**trp53**	Forward primer	GACAGCAGGGCTCACTCCAGCTAC
	Reverse primer	GGACGGGATGCAGAGGCAGTCA
**p21Waf1**	Forward primer	TGGTGTCTGAGCGGCCTGAAGA
	Reverse primer	TCCCGTGGGCACTTCAGGGT
**PUMA**	Forward primer	GAGCGGCGGAGACAAGAAGAGC
	Reverse primer	AGGATCCCTGGGTAAGGGGAGGAG
**PHLDA3**	Forward primer	CGCCACATCTACTTCACGCT
	Reverse primer	CACAGTCTGGATGGCCTGTT
**CHOP**	Forward primer	GAGCTGGAAGCCTGGTATGA
	Reverse primer	ACGCAGGGTCAAGAGTAGTG
**ISG15**	Forward primer	GACTCCTTAATTCCAGGGGACC
	Reverse primer	CACCAGGAAATCGTTACCCCC
**NLRC5**	Forward primer	CCTTTGGACATGGAGATACCAC
	Reverse primer	GTGAGGACCCATAGCTCTGAA
**TNFRSF1A**	Forward primer	AGAGAAAGTGAGTGCGTCCC
	Reverse primer	TGTGACATTTGCAAGCGGAG
**DRAM1**	Forward primer	CTCTCTGGGCACGTCAACC
	Reverse primer	TCTGGAGGAGTTGTTCCTGTGT
**B-Actin**	Forward primer	CAACGAGCGGTTCCGATG
	Reverse primer	GCCACAGGATTCCATACCCA
**GAPDH**	Forward primer	CAGCAACTCCCACTCTTCC
	Reverse primer	CCATGTAGGCCATGAGGTTC

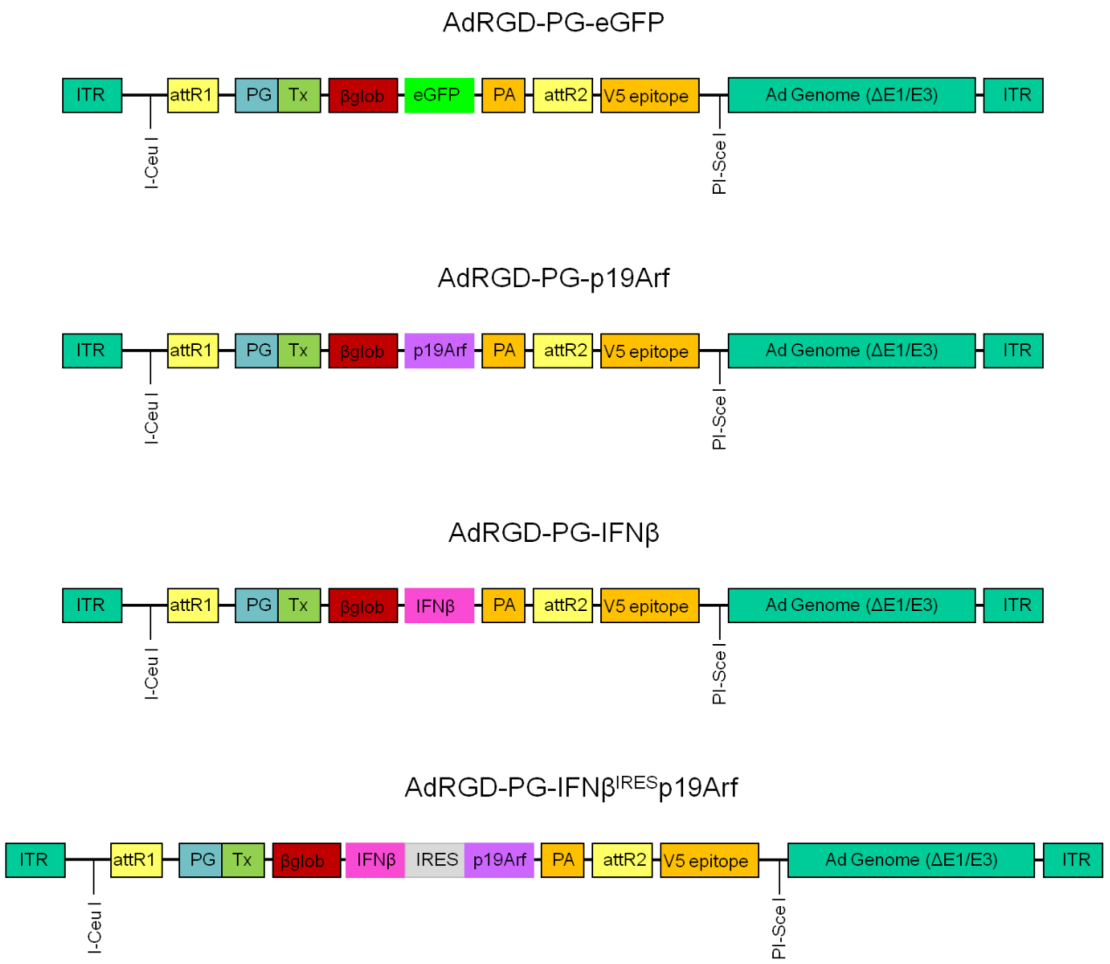

Figure S1: Schematic representation of the AdRGD-PG vectors. ITR: adenoviral replicative inverted terminal repeats; attR1 and attR2: site-specific recombination sequences; PG/Tx/βglob: p53-responsive promoter, composed by PG enhancer sequence, TATA box (Tx) and βglobin (βglob) intronic sequences;; eGFP: enhanced green fluorescent protein cDNA sequence; p19Arf: p19Arf cDNA sequence; IFNβ: mouse interferon-β cDNA sequence; IRES: internal ribosome entry site sequence; PA: polyadenylation sequence; V5 epitope: V5 epitope of adenoviral capsid sequence; ΔE1/E3: deletion of genes E1 and E3.

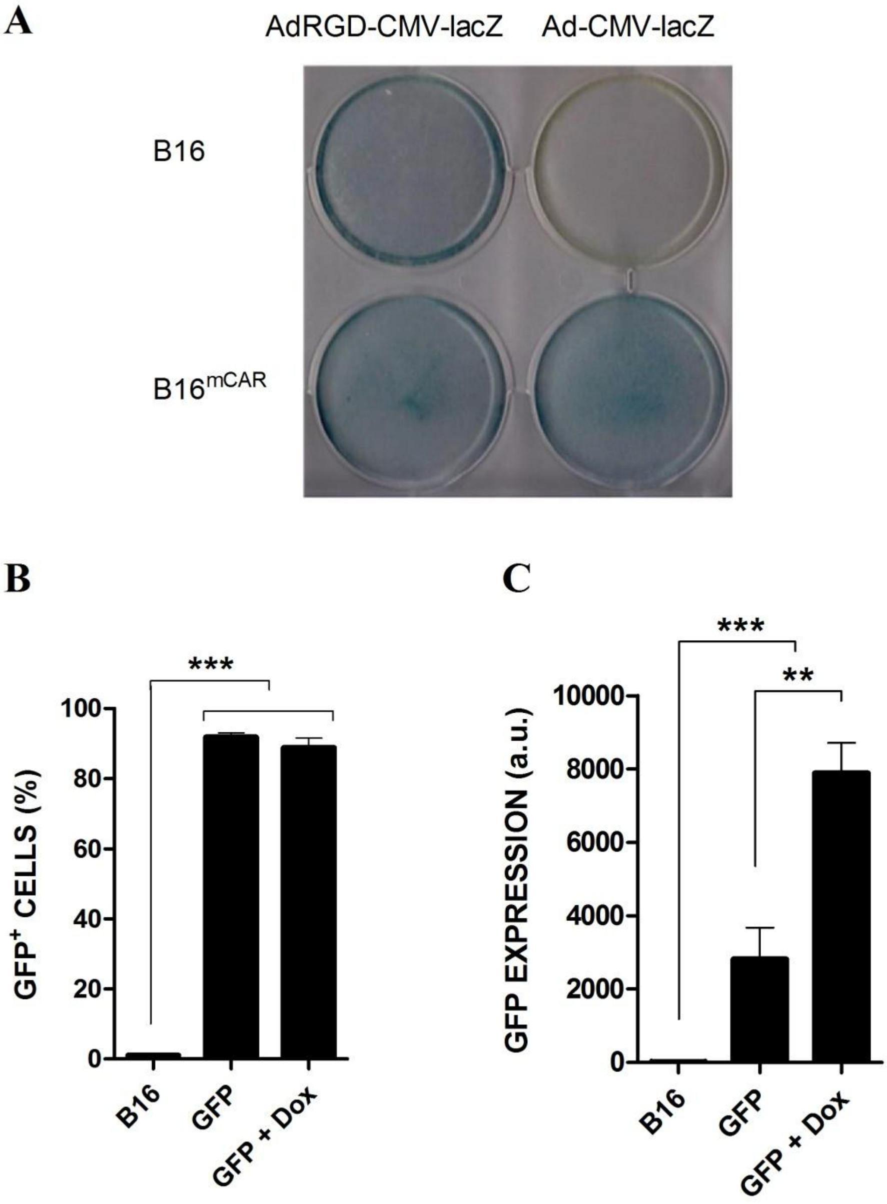

Figure S2: RGD-modified vector efficiently transduces CAR-negative cells. (A) Parental B16 and B16mCAR (with forced expression of murine CAR) were transduced under identical conditions and later stained to reveal β-galactosidase activity. **(B)** B16 cells were transduced with AdRGD-PG-eGFP (MOI=50), treated or not with 100 ng/ml doxorubicin (+Dox) and the percentage of eGFP-positive cells was measured by flow cytometry. **(C)** The relative expression (intensity) of eGFP is given in arbitrary units (au), as determined by flow cytometry. Results from each graph represent the average and s.d. from three independent experiments. **P<0.01, ***P<0.001, one-way analysis of variance, followed by Tukey's Multiple Comparison.

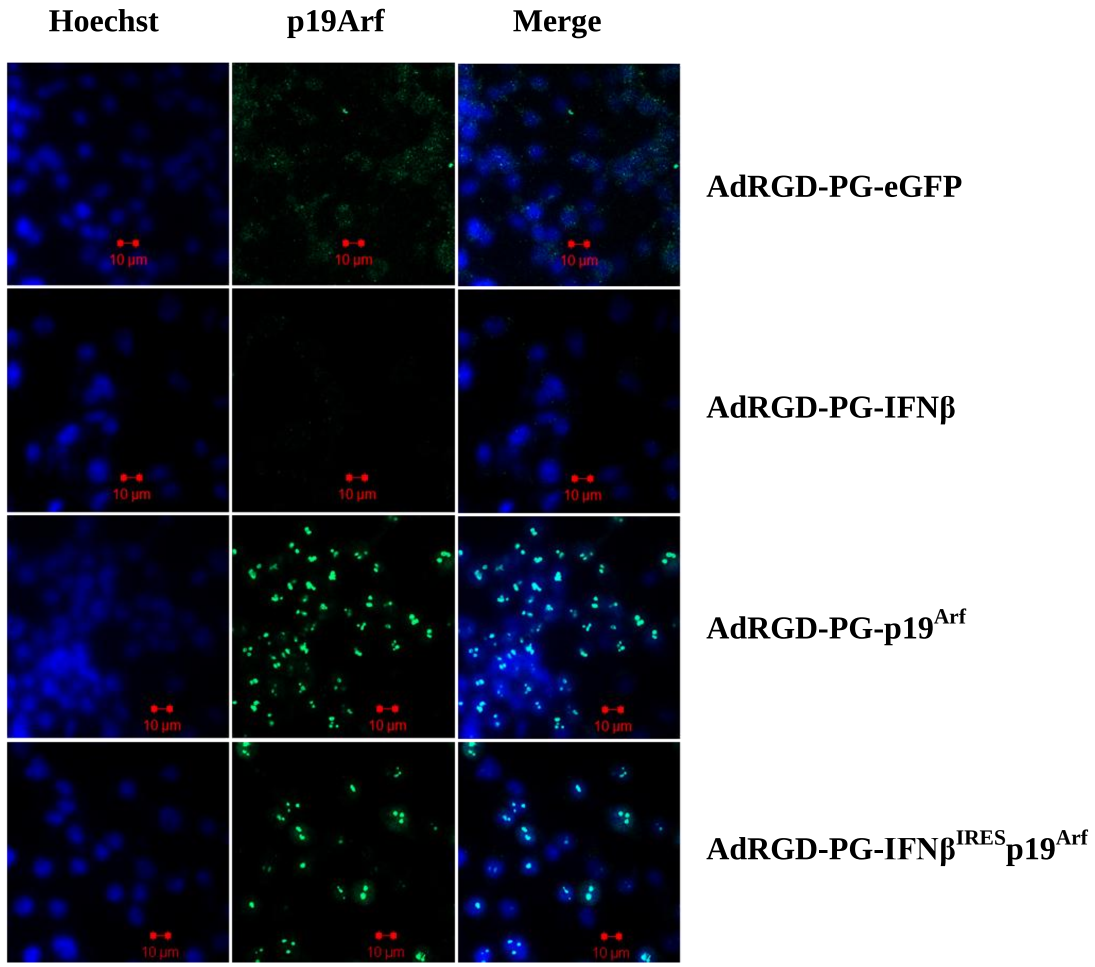

Figure S3: Reliable p19Arf transgene expression from the mono- and bicistronic AdRGD-PG vectors. Exogenous p19Arf detected by immunofluorescence. B16 cells were transduced with a MOI of 25 with the indicated vectors and, 48 hours later, fixed with methanol and exposed to a polyclonal antibody for p19Arf prior to an Alexa-488 anti-rabbit secondary antibody. Cells were then stained with Hoechst 33258. Merge refers to the combined images of the green and blue channels.

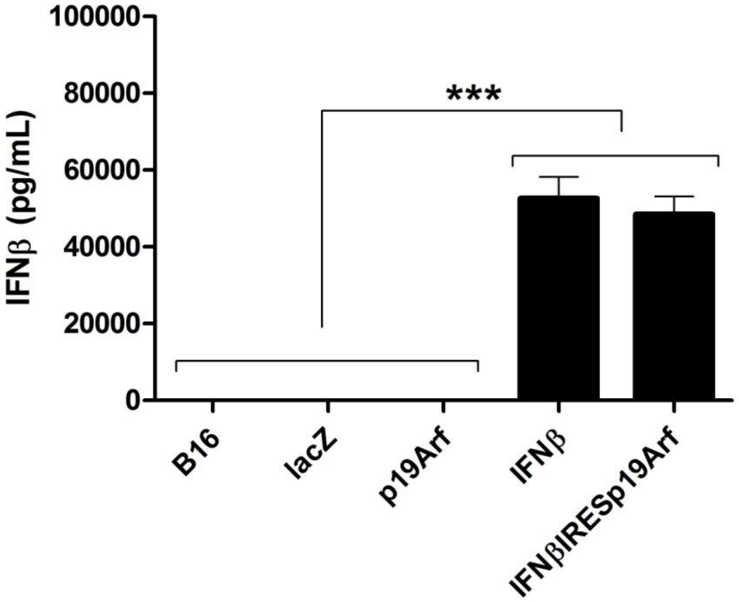

Figure S4: Reliable IFNβ transgene expression from the mono- and bicistronic AdRGD-PG vectors. B16 cells were transduced with a multiplicity of infection (MOI) of 25 with the indicated vectors and the medium where B16 were cultivated was collected 48 hours later for detection of IFNβ by ELISA. Results from each graph represent the average and s.d. from three independent experiments. ****P*<0.001, one-way analysis of variance, followed by Tukey's Multiple Comparison.

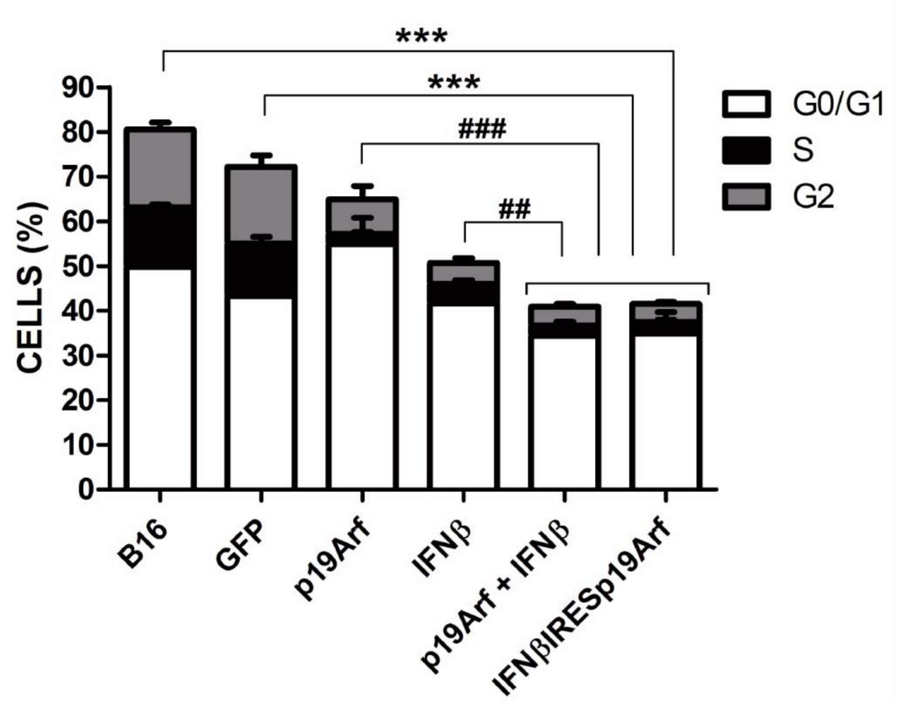

Figure S5: Gene transfer of p19Arf and IFNβ alters cell cycle distribution. B16 cells were transduced with the AdRGD-PG vector encoding the indicated transgene (MOI 100) and cell cycle distribution at 48 hours was assessed. Results represent the average and s.d. from three independent experiments. ****P*<0.001 for all cell cycle phases, ##*P*< 0.005 for G0/G1 phase, ###*P*<0.001 for G0/G1 phase, two-way analysis of variance, followed by Bonferroni.

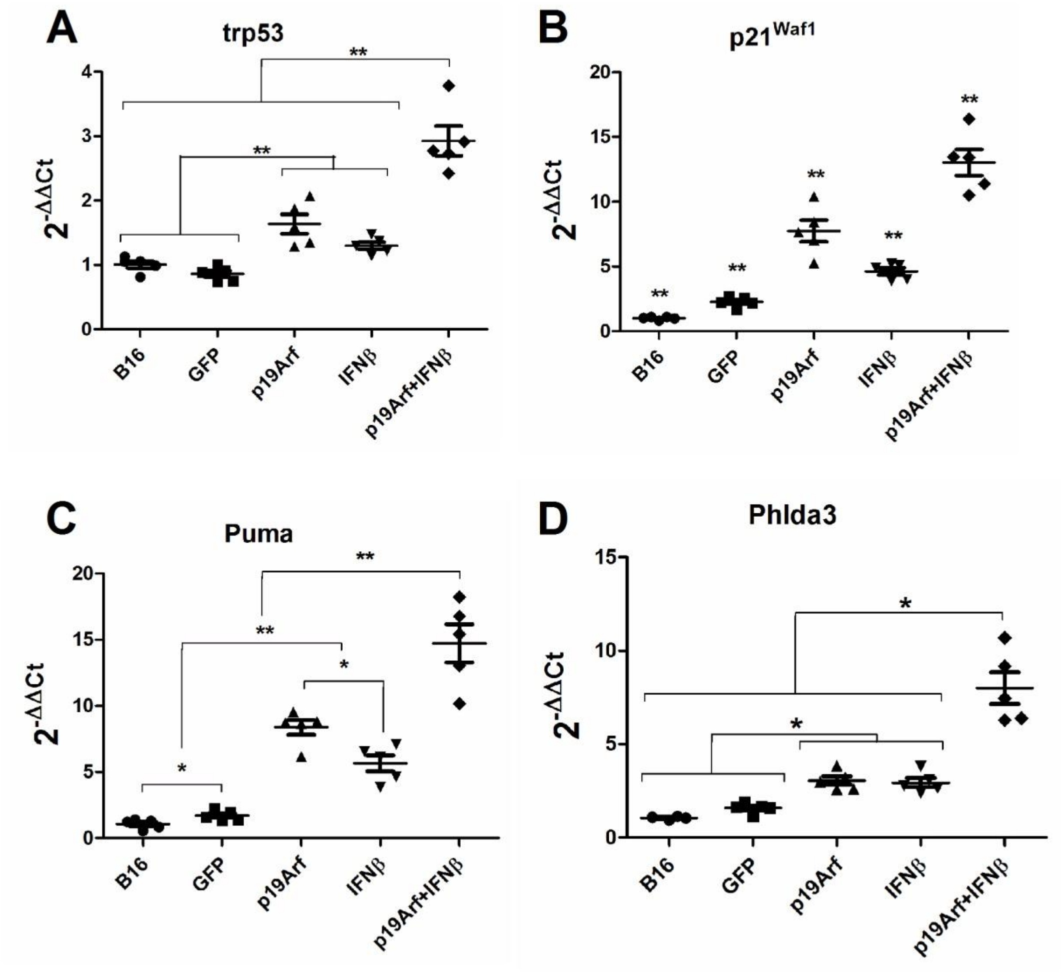

Figure S6: Gene transfer of p19Arf + IFNβ induces trp53 pathway *in vitro*. Cells were transduced with AdRGD-PG vectors and incubated for 36 hours before total RNA extraction and RT-qPCR analysis of p53 pathway genes (Trp53, p21Waf1 (Cdkn1a), PUMA, PHLDA3). β-Actin and/or glyceraldehyde 3-phosphate dehydrogenase (GAPDH) were used as the reference genes. Data represents the average and s.d. from duplicated PCR reactions derived from 5 independent biological experiments. *P<0.05, **P<0.01 one-way analysis of variance, followed by Kruskal-Wallis.

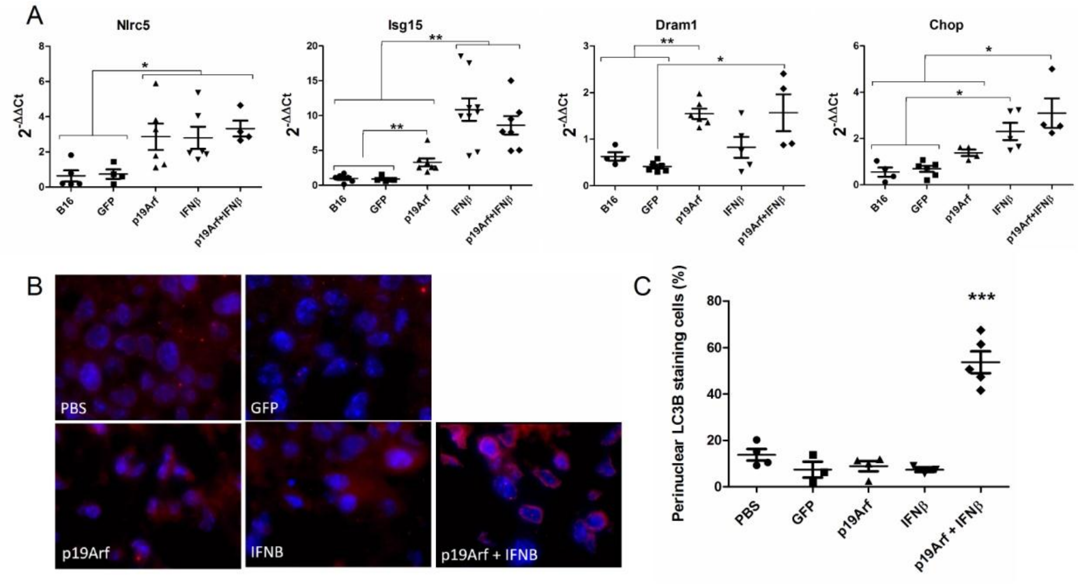

Figure S7: *In situ* gene therapy with p19Arf + IFNβ induces expression of antiviral response genes. Mice were inoculated with $1x10^6$ B16 cells in the left flank, treated with 3 injections of $5x10^8$ infectious units (i.u.) of AdRGD vectors every two days and tumors were collected 1 day after the last injection. **(A)** Total RNA was extracted and RT-qPCR analysis was performed for antiviral response genes (NLRC5 and ISG15), DRAM1 and CHOP. β-Actin and/or glyceraldehyde 3-phosphate dehydrogenase (GAPDH) were used as the reference genes. Data represents the average and s.d. from duplicated PCR reactions derived from 7-10 animals per group. *P<0.05 and **P<0.01, one-way analysis of variance, followed by Kruskal-Wallis. **(B)** Immunofluorescence detection of LC3β in frozen sections and **(C)** quantification of cells showing vesicular, perinuclear LC3β expression. Data represents the average and s.d. from 7-10 animals per group. ***P<0.001, one-way analysis of variance, followed by Tukey's Multiple Comparison.

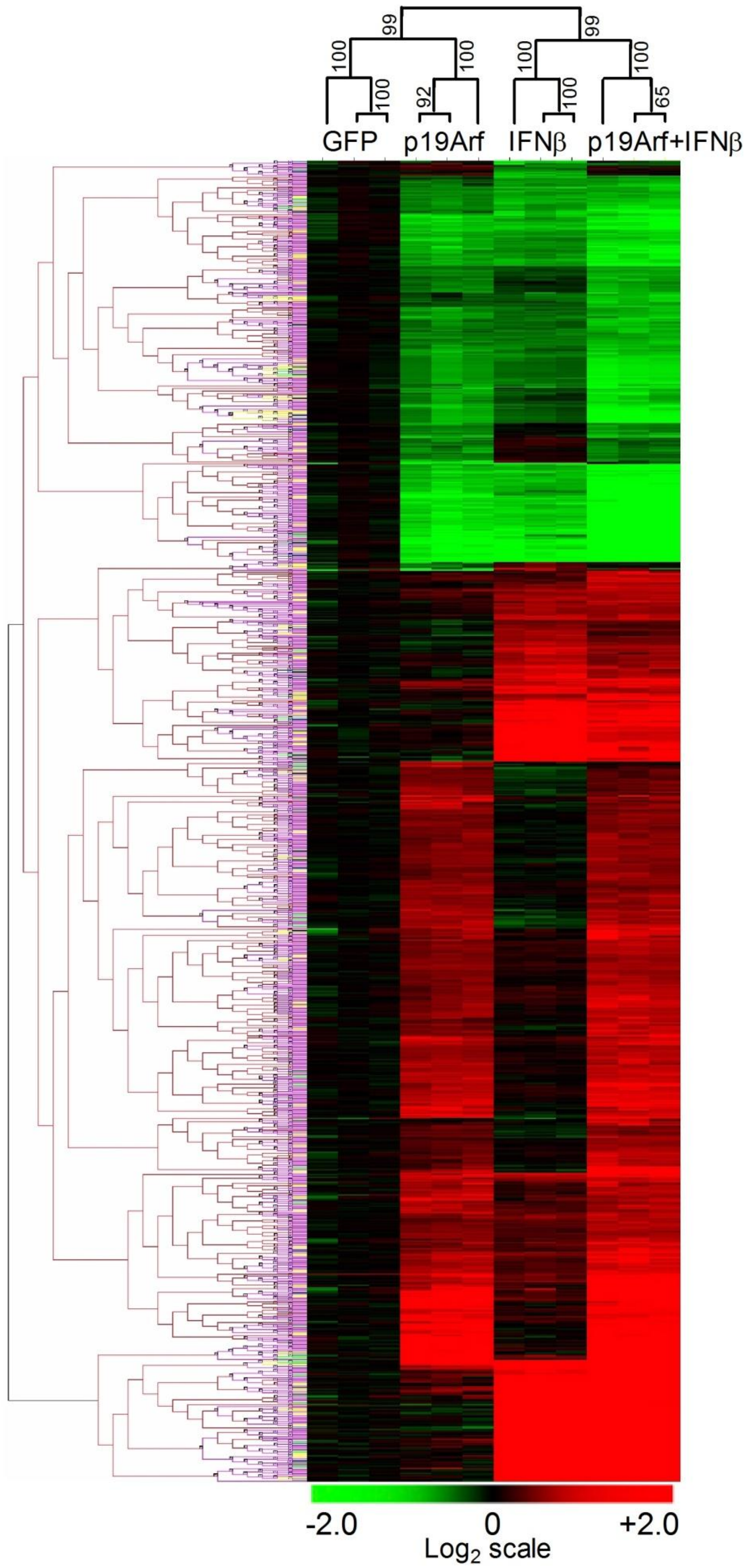

Figure S8: Hierarchical cluster was designed using Euclidian distance and complete linkage. Bootstrap was used to evaluate dendogram's consistency.
