# Supplementary material for: Reestablishment of p53/Arf and interferon-β pathways mediated by a novel adenoviral vector potentiates antiviral response and immunogenic cell death
Source: Cell Death Discov. 2017 Mar 20;3:17017–. doi: 10.1038/cddiscovery.2017.17 (PMC5357668; doi:10.1038/cddiscovery.2017.17)
Supplement: Supplementary Information [file cddiscovery201717-s6.pdf]

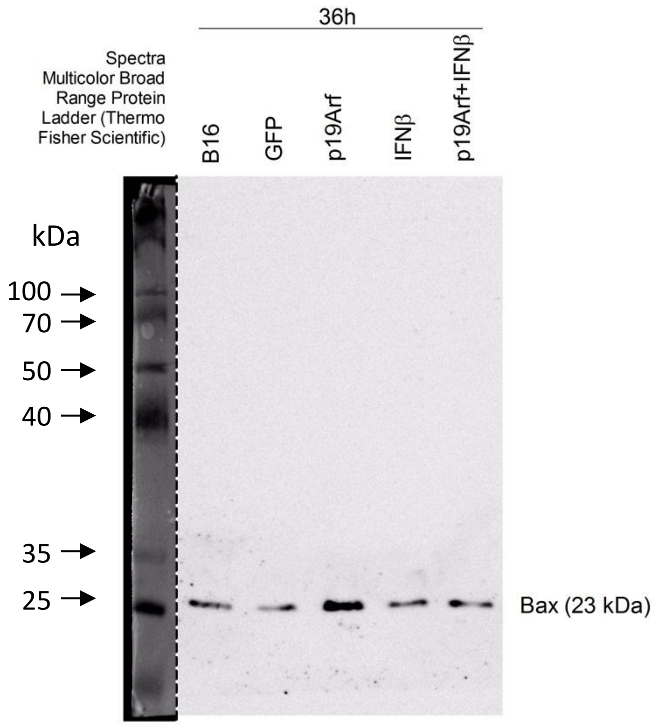

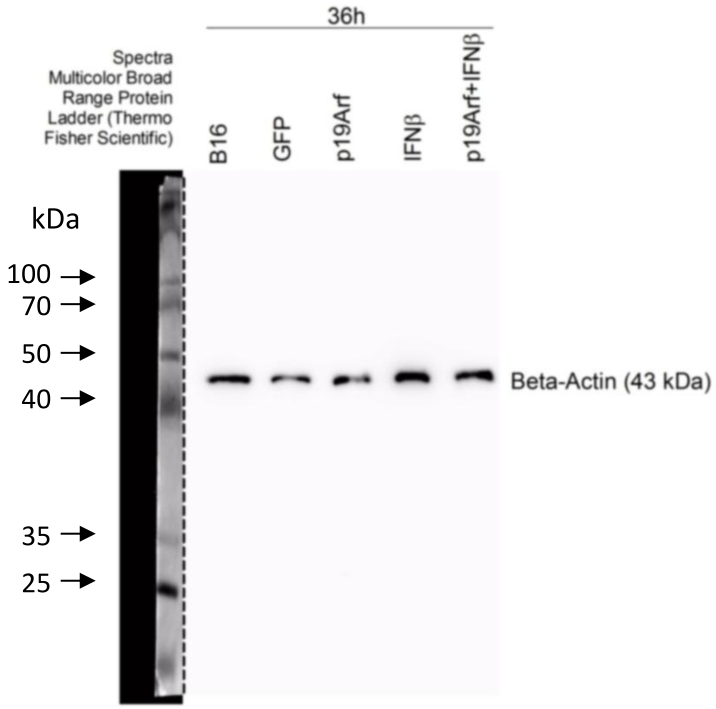

Complete images, western blot shown in Figure 4. Note that the pre-stained size marker does not react upon detection of the specific protein. We show the ladder imaged in the gel and the blot after detection of the specific protein.

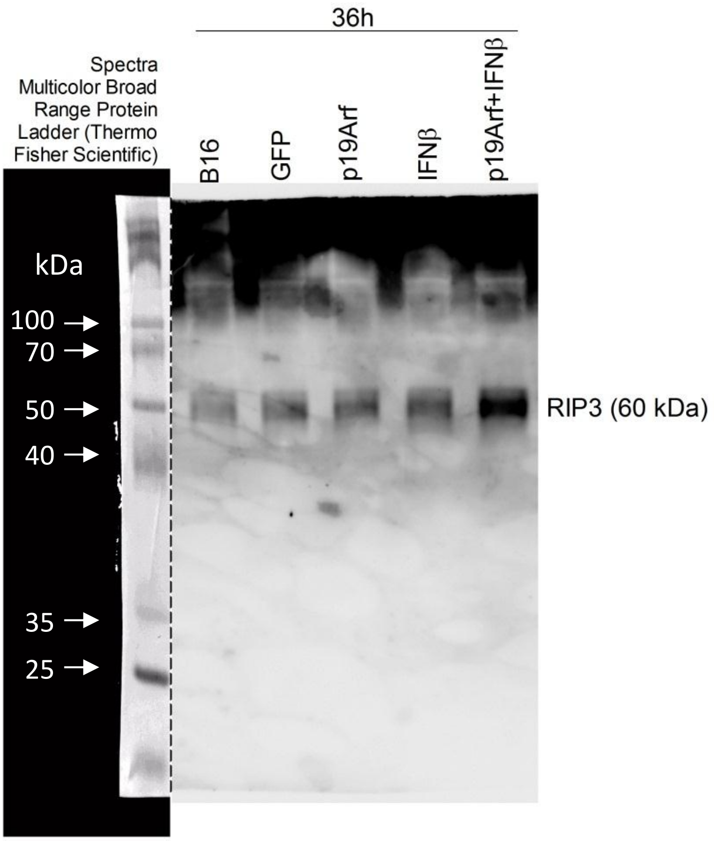

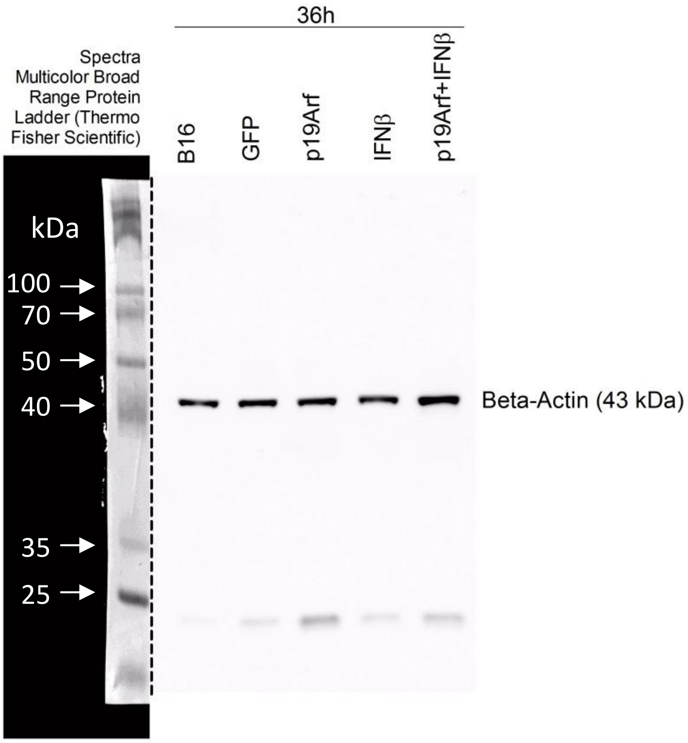

Complete images, western blot shown in Figure 5. Note that the pre-stained size marker does not react upon detection of the specific protein. We show the ladder imaged in the gel and the blot after detection of the specific protein.
